# Supplementary material for: Unsupervised learning reveals landscape of local structural motifs across protein classes
Source: Bioinformatics. 2025 Jun 26;41(7):btaf377. doi: 10.1093/bioinformatics/btaf377 (PMC12258146; doi:10.1093/bioinformatics/btaf377)
Supplement: btaf377_Supplementary_Data [file btaf377_supplementary_data.zip › Supplementary_material_PDF.pdf]

Supplementary materials

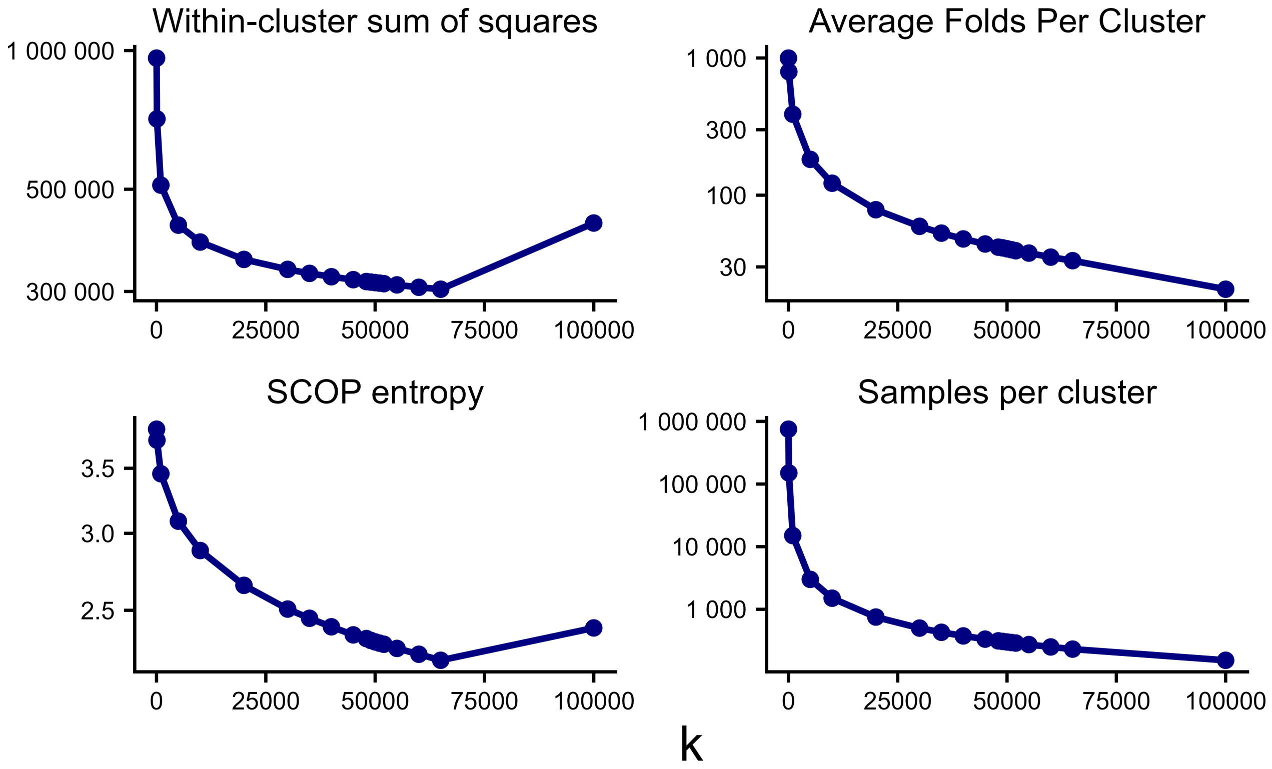

Figure S1. Cluster characteristics at varying *k*. Clockwise from top left: (1) within-cluster sum of squares, (2) unique SCOP folds per cluster, (3) entropy of SCOP families within each cluster, and (4) average number of environments in each cluster.

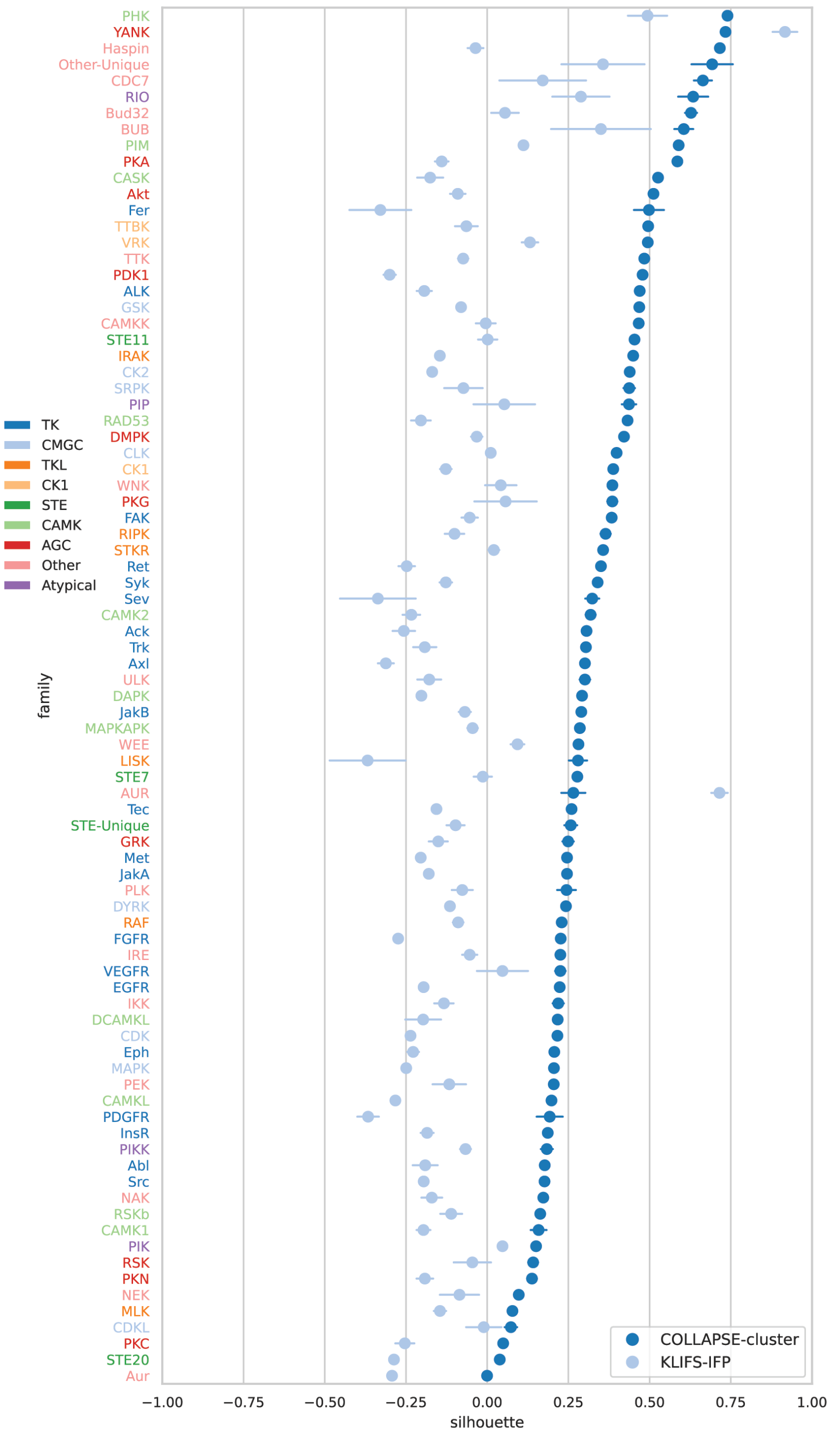

Figure S2. Silhouette scores of pocket similarity for all 95 kinase families. Each row represents a single kinase family, and the dots represent the mean silhouette score over all kinase pockets within that family. Errorbars represent the standard error of the mean. Row labels are colored by the high-level kinase group that each family belongs to.

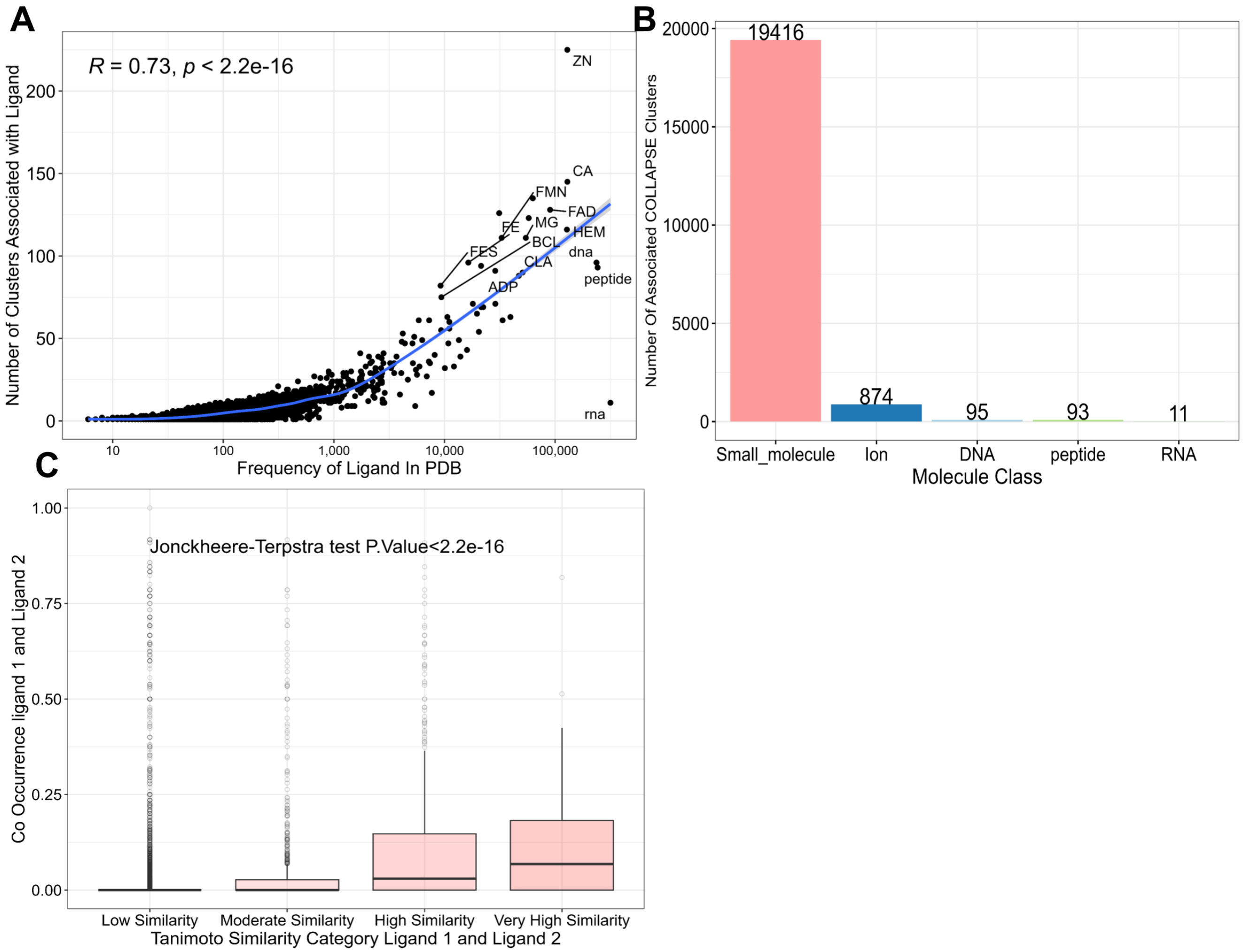

Figure S3. **A.** Identification of ligands which are commonly associated with specific structural motifs, showing a correlation between how common a ligand is in PDB, and how commonly its associated with structural motifs, with specific ligands such as DNA, RNA, peptides, and ions being outliers to this correlation (Spearman correlation). **B.** Number of COLLAPSE clusters Associated with each molecular class. **C.** Distribution of molecular classes across ligand specificity categories of COLLAPSE clusters. Each ligand specificity category is defined by the proportion of residues within the COLLAPSE clusters that bind a specific ligand. **D.** Distribution of normalized co-occurrence scores for ligand-ligand pairs categorized by Tanimoto similarity (See methods 2.7).

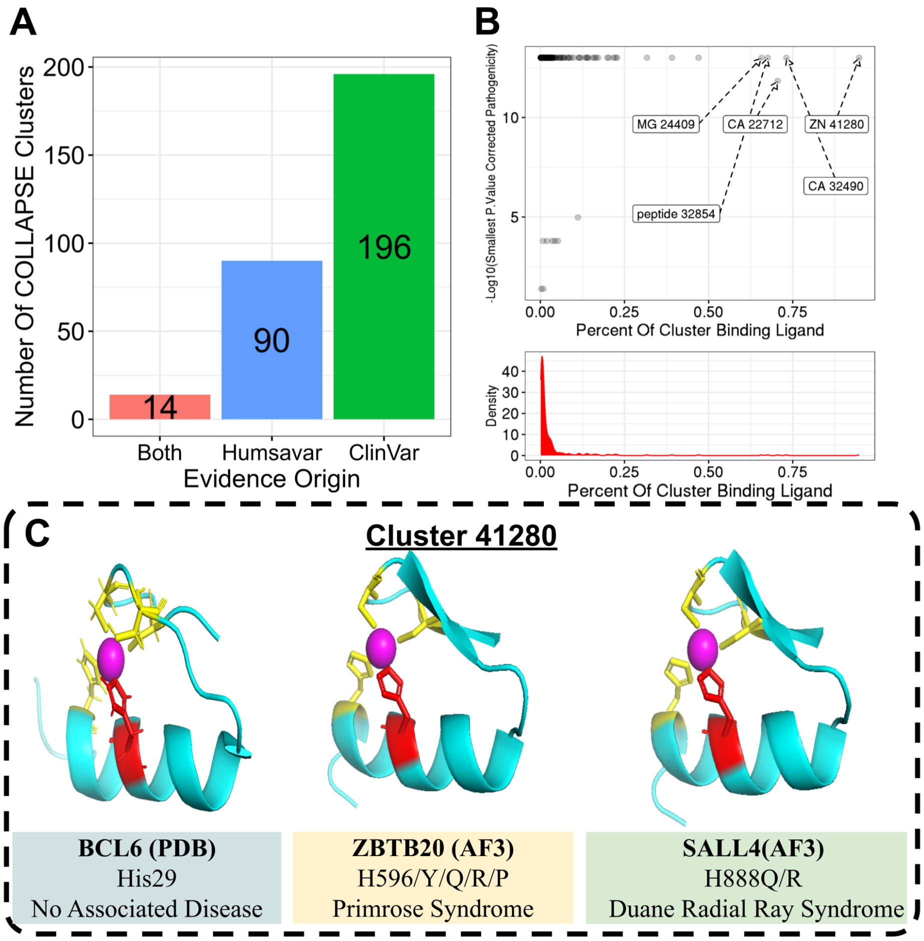

Figure S4. **A.** Number of COLLAPSE clusters associated with human diseases as indicated by data from Humsavar, ClinVar, and from both. **B.** Top: Each COLLAPSE cluster ($n = 92$ clusters) that are both pathogenic and exhibit an enrichment for residues that bind ligands in the PDB. The y-axis represents the $-\log_{10}$ of the smaller p-value, derived from either Humsavar or ClinVar, and the x-axis represents the proportion of residues within the cluster that bind the ligand. Bottom: Distribution of the pathogenic ligand binding collapse clusters' ligand specificity. **C.** Cluster 41280, which is centered around a histidine (red) coordinating a zinc ion along with additional cysteines and histidines (yellow).

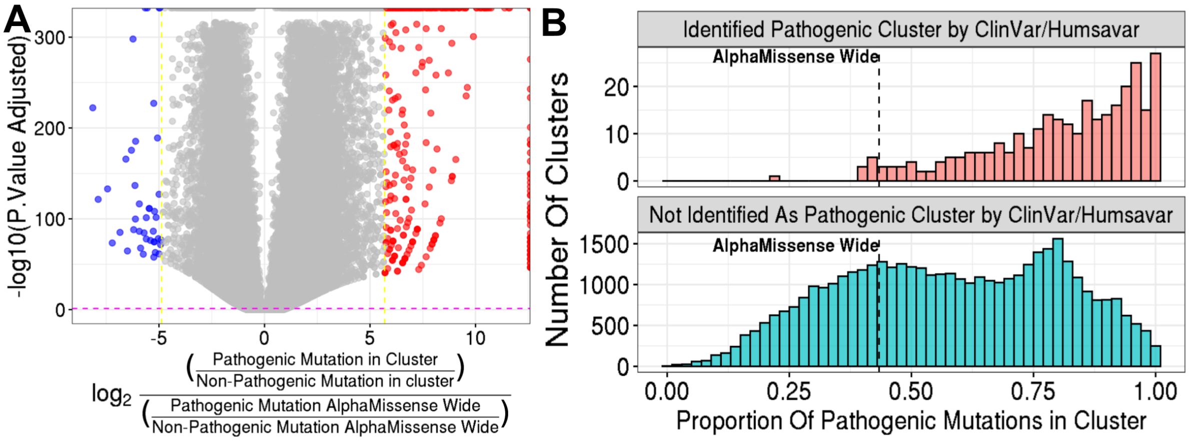

Figure S5. **A.** Volcano plot of all COLLAPSE clusters found in humans, each point representing an individual COLLAPSE cluster's multiple testing adjusted p.value derived from a hypergeometric test to identify enrichment of pathogenic variants, as well as the proportion of pathogenic variants in the cluster compared to the proportion found AlphaMissense Wide. Red dots are clusters with an enrichment of pathogenic variants, blue dots are clusters with a depletion of pathogenic variants. Some clusters have an infinite value in the x axis, meaning any mutation is pathogenic in the cluster. **B.** Histograms representing the proportion of pathogenic mutations in each cluster. We see that the vast majority of the 300 ClinVar/Humsavar pathogenic clusters have a higher proportion of pathogenic mutations then AlphaMissense Wide.

Table S1. Kinase inhibitors evaluated in this study, as well as their primary target(s) as specified by Karaman *et al.* [30].

Inhibitor name	Alternative name (Karaman *et al.*)	Primary target(s)
Erlotinib		EGFR
Pelitinib	EKB-569	EGFR
Vandetanib	ZD-6474	EGFR, RET
Dasatinib		ABL1, SRC
Sunitinib		KIT, FLT3
Pazopanib	GW-786034	FLT1, FLT4
Midostaurin	PKC-412	FLT3, KIT
Ruboxistaurin	LY-333531	PKCb
Tozasertib	VX-680/MK-0457	AurA, AurB, AurC

Table S2. Spearman rank correlation between fingerprint similarity and experimental binding affinity for each kinase inhibitor-target pair. Bold indicates the best correlation for each pair, and stars represent Bonferroni-corrected statistical significance: *** ($p < 0.01$), ** ($p < 0.05$), * ($p < 0.1$).

Inhibitor	Target	COLLAPSE-cluster	KLIFS-IFP
Dasatinib	ABL1	**0.529*** **	0.464***
	SRC	**0.533*** **	0.289
Erlotinib	EGFR	0.141	**0.356**
Pelitinib	EGFR	-0.040	**0.180**
Midostaurin	FLT3	0.013	**0.083**
	KIT	**0.092**	-0.084
Sunitinib	FLT3	**0.348*** **	0.177
	KIT	**0.357*** **	0.060
Tozasertib	AurA	**0.215**	-0.018
	AurB	0.332**	**0.337** **
	AurC	**0.327** **	0.280
Vandatenib	EGFR	0.148	**0.168**
Pazopanib	FLT1	**0.659*** **	0.460**
Ruboxistaurin	PKCb	0.250	**0.284**

Table S3. Protein metadata for all mutations in Humsavar associated with cluster 6044, organized by Uniprot ID. Asterisks (*) indicate non-pathogenic mutations.

Uniprot ID	Protein name	Gene name	Mutations	Diseases
O75096	Low-density lipoprotein receptor-related protein 4	LRP4	C1017R	Cenani-Lenz syndactyly syndrome (CLSS)
P00740	Coagulation factor IX	F9	C97S, C108S, C134Y	Hemophilia B (HEMB)
P01130	Low-density lipoprotein receptor	LDLR	C318Y*, C318F, C318R, C329Y, C329F, C358Y, C364R*, C368R, C368Y*	Hypercholesterolemia, familial, 1 (FHCL1)
P07225	Vitamin K-dependent protein S	PROS1	C121Y, C161G, C175F, C247G	Thrombophilia due to protein S deficiency, autosomal dominant (THPH5)
P07911	Uromodulin	UMOD	C77Y, C126R, C170Y, C300G, C315R	Tubulointerstitial kidney disease, autosomal dominant, 1 (ADTKD1)
P08709	Coagulation factor VII	F7	C121F, C151S	Factor VII deficiency (FA7D)
P23352	Anosmin-1	ANOS1	C163Y, C163R, C172R	Hypogonadotropic hypogonadism 1 with or without anosmia (HH1)
P24043	Laminin subunit alpha-2	LAMA2	C527Y	Merosin-deficient congenital muscular dystrophy 1A (MDC1A)
P35555	Fibrillin-1	FBN1	C504F, C504R, C546W, C576Y, C587Y, C727Y, C781R, C781Y, C811Y, C926R, . . . , [46 more]	Marfan syndrome (MDS); Ectopia lentis 1, isolated, autosomal dominant (ECTOL1)
P35556	Fibrillin-2	FBN2	C1246F, C1257W, C1257R, C1384F, C1384Y, C1253Y, C1253W, C1257W, C1257R, C1268R, C1253Y, C1253W	Contractural arachnodactyly, congenital (CCA)
P46531	Neurogenic locus notch homolog protein 1	NOTCH1	C429R	Adams-Oliver syndrome 5 (AOS5)
P78504	Protein jagged-1	JAG1	C911Y	Alagille syndrome 1 (ALGS1)
P82279	Protein crumbs homolog 1	CRB1	C310Y, C891G, C939Y, C1181R,C1223S	Retinitis pigmentosa 12 (RP12); Leber congenital amaurosis 8 (LCA8);
Q5IJ48	Protein crumbs homolog 2	CRB2	C620S	Focal segmental glomerulosclerosis 9 (FSGS9)
Q5T1H1	Protein eyes shut homolog	EYS	C1176R	Retinitis pigmentosa 25 (RP25)
Q6UXH8	Collagen and calcium-binding EGF domain-containing protein 1	CCBE1	C102S	Hennekam lymphangiectasia-lymphedema syndrome 1 (HKLLS1)
Q92832	Protein kinase C-binding protein NELL1	NELL1	C553F*	N/A
Q9HC23	Prokineticin-2	PROK2	C46Y	Hypogonadotropic hypogonadism 4 with or without anosmia (HH4)

Table S4. Protein metadata for all mutations in Humsavar associated with cluster 31037, organized by Uniprot ID. Asterisks (*) indicate non-pathogenic mutations.

Uniprot ID	Protein name	Gene name	Mutations	Diseases
O15118	NPC intracellular cholesterol transporter 1	NPC1	D948N, D948H, D948Y	Niemann-Pick Disease C1 (NPC1)
O95528	Solute carrier family 2, facilitated glucose transporter member 10	SLC2A10	E437K	Arterial tortuosity syndrome (ATORS)
P11166	Solute carrier family 2, facilitated glucose transporter member 1	SLC2A1	E329Q	GLUT1 deficiency syndrome 1 (GLUT1DS1)
P26440	Isovaleryl-CoA dehydrogenase, mitochondrial	IVD	D72N	Isovaleric acidemia (IVA)
P35520	Cystathionine beta-synthase	CBS	D234N	Cystathionine beta-synthase deficiency (CBSD)
P51531	Probable global transcription activator SNF2L2	SMARCA2	D1158V	Nicolaides-Baraitser syndrome (NCBRS)
Q7Z3K3	Pogo transposable element with ZNF domain	POGZ	E1040K	Autism spectrum disorder (ASD)
Q9NRW7	Vacuolar protein sorting-associated protein 45	VPS45	E238K	Neutropenia, severe congenital 5, autosomal recessive (SCN5)
Q9Y5Z9	UbiA prenyltransferase domain-containing protein 1	UBIAD1	D236E	Corneal dystrophy, Schnyder type (SCCD)

Table S5. Protein metadata for all mutations in Humsavar associated with cluster 23310, organized by Uniprot ID. Asterisks (*) indicate non-pathogenic mutations.

Uniprot ID	Protein name	Gene name	Mutations	Diseases
P04629	High affinity nerve growth factor receptor	NTRK1	Y359C	Congenital insensitivity to pain with anhidrosis (CIPA)
P11362	Fibroblast growth factor receptor 1	FGFR1	Y99C, Y228D, Y339C	Hypogonadotropic hypogonadism 2 with or without anosmia (HH2)
P21802	Fibroblast growth factor receptor 2	FGFR2	Y105C, Y340H, Y340C	Crouzon syndrome (CS); Pfeiffer syndrome (PS)
Q0ZGT2	Nexilin	NEXN	Y652C	Cardiomyopathy, dilated, 1CC (CMD1CC)
Q14896	Myosin-binding protein C, cardiac-type	MYBPC3	Y237S	Cardiomyopathy, familial hypertrophic, 4 (CMH4)
Q86TC9	Myopalladin	MYPN	Y339F	Cardiomyopathy, dilated, 1KK (CMD1KK)
